# 1066. *In Vitro and in Vivo* Antimicrobial Activity of Cefiderocol and Comparators against *Achromobacter* spp

**DOI:** 10.1093/ofid/ofab466.1260

**Published:** 2021-12-04

**Authors:** Ryuichiro Nakai, Ayaka makino, Hitomi Hama, Toriko Yoshitomi, Rio Nakamura, Meredith Hackel, Miki Takemura, Daniel F Sahm, Yoshinori Yamano

**Affiliations:** 1 Shionogi TechnoAdvance Research & Co., Ltd., Toyonaka, Osaka, Japan; 2 Shionogi & Co., Ltd., Osaka, Osaka, Japan; 3 IHMA, Inc., Schaumburg, Illinois

## Abstract

**Background:**

*Achromobacter* spp. is intrinsically resistant to multiple antibiotics, and the treatment options are limited. Cefiderocol (CFDC), a siderophore cephalosporin approved in US and EU, is active against a wide variety of aerobic Gram-negative bacteria, including carbapenem-resistant strains. In this study, *in vitro* and *in vivo* antibacterial activity of CFDC against *Achromobacter* spp. was evaluated.

**Methods:**

A total of 334 global isolates collected by IHMA from 39 countries in 2015-2019 were used. Minimum inhibitory concentrations (MICs) of CFDC and comparators were determined by broth microdilution method using iron-depleted CAMHB or CAMHB, respectively, as recommended by CLSI guidelines. *In vivo* efficacy of CFDC was compared with meropenem (MEM), piperacillin-tazobactam (PIP/TAZ), ceftazidime (CAZ), and ciprofloxacin (CIP) in a neutropenic murine lung infection model (n=5), and compared with MEM in a immunocompetent rat lung infection model (n=3-7) caused by 2 *A. xylosoxydans*. In the murine model, treatment was given 2, 5, and 8 hours post-infection, and the numbers of viable cfu in lungs were determined 24 hours post-infection. In the rat model, the humanized PK in plasma resulting from CFDC 2 g every 8 h (3-h infusion) or meropenem 1 g every 8 h (0.5-h infusion) were recreated via continuous intravenous infusion for 4 days, following which cfu in lungs were determined.

**Results:**

CFDC showed *in vitro* activity with MIC_50/90_ of 0.06/0.5 µg/mL against 334 *Achromobacter* spp. Only 7 isolates (2.1%) had MICs > 4 µg/mL. These were the lowest values among all compound tested (Table). In the murine model, CFDC caused > 1.5 log_10_ decrease of viable cfu in lungs at 100 mg/kg dose (%*f*T >MIC: < 50%) from baseline control against both of strains (CFDC MIC: 0.5 and 2 µg/mL) (*P*< 0.05). No decrease of cfu in lungs was observed for the comparators at 100 mg/kg (MEM, PIP/TAZ, CAZ, and CIP MICs were >16, >64, >32, and >8 µg/mL, respectively). In the rat model, humanized CFDC dosing reduced the viable cfu by >1 log_10_ CFU/lung compared with baseline controls (*P*< 0.05). MEM showed no significant activity.

In vitro activity of CFDC and comparator agents against Achromobacter spp.

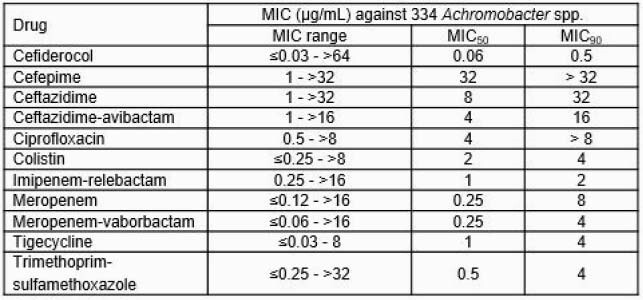

334 Achromobacter spp. isolates collected from 2015 and 2019. The majority of isolates tested were A. xylosoxidans (312/334; 93.4%), followed by A. insolitus (11/334; 3.3%), Achromobacter sp. (8/334; 2.4%), A. denitrificans (2/334; 0.6%), and A. piechaudii (1/334; 0.3%).

**Conclusion:**

CFDC showed potent *in vivo* efficacy reflecting *in vitro* activity against *A. xylosoxidans.* The results suggested that CFDC has the potential to be an effective therapeutic option for *Achromobacter* spp. infections.

**Disclosures:**

**Ryuichiro Nakai, MSc**, **Shionogi TechnoAdvance Research & Co., Ltd.** (Employee) **Ayaka makino, BSc**, **Shionogi TechnoAdvance Research & Co., Ltd.** (Employee) **Toriko Yoshitomi,** -, **Shionogi TechnoAdvance Research & Co., Ltd.** (Employee) **Rio Nakamura, BSc**, **Shionogi TechnoAdvance Research & Co., Ltd.** (Employee) **Meredith Hackel, PhD MPH**, **IHMA** (Employee)**Pfizer, Inc.** (Independent Contractor) **Miki Takemura, MS**, **SHIONOGI & CO., LTD.** (Employee) **Daniel F. Sahm, PhD**, **IHMA** (Employee)**Pfizer, Inc.** (Independent Contractor) **Yoshinori Yamano, PhD**, **Shionogi** (Employee)

